# Multimorbidity: Addressing the next global pandemic

**DOI:** 10.1371/journal.pmed.1004229

**Published:** 2023-04-04

**Authors:** The PLOS Medicine Editors

**Affiliations:** Public Library of Science, San Francisco, California, United States of America and Cambridge, United Kingdom

Advances in translational scientific research and modern medicine have enabled substantial progress to be made in clinical practices around the world. And, while there are no doubts that inequities persist, these advances have contributed to significant improvements in life expectancy globally. It may come as no surprise, considering the advances made, that the number of people being diagnosed as multimorbid—with two or more chronic conditions, either physical, mental or both—has also progressively increased.

In low- and middle-income countries (LMICs) the problem is ever more palpable. Plagued by inequitable access to research facilities, healthcare services, diagnostics, therapeutics and effective policy implementation, progress lags far behind that of high-income countries (HICs). Not only is there a disproportionately high prevalence of chronic multisystem infectious diseases, such as HIV and TB, the prevalence of non-communicable conditions, such as hypertension and diabetes, have also increased over the preceding decades [1].

Historically, in many HICs multimorbidity has been attributed to advancing age [2]. While this has been and will continue to be one of the primary drivers of multimorbidity, other socioeconomic and structural factors—such as income, education, and location—contribute significantly to an individual’s likelihood of being diagnosed with multiple conditions. As a result, in socioeconomically deprived regions, multimorbidity is becoming increasingly evident in younger populations [3]. The emergence of COVID-19 and the consequence now known as “long-COVID” which affects multiple organ systems simultaneously, as well as numerous other conditions, such as HIV infection, multisystem autoimmune conditions, cancer, diabetes, and mental ill-health have further highlighted the age indiscriminate nature of multimorbidity.

How individual conditions should be managed in tandem with others, and by whom, is often at the heart of the debate when putting the theoretical management of multimorbid patients into practice. It is frequently the case that, at least in the initial stages of multimorbidity manifestation, the contribution of each individual condition is minimal or limited while the cumulative effect of the conditions combined pose a major problem, not only to the patient but also to individual caregivers. Medical anthropologists have proposed the theory of “syndemics” in which diseases are hypothesized to cluster and to interact with one another (the biological–biological interface) as well as with social and structural factors (the biological–social interface) [4]. This clustering of conditions is often considered predictable, for example obesity, diabetes, and hypertension. However, syndemic clusters have usually been observed in inductive studies, and empirical research has revealed unexpected clustering of chronic conditions, such as the extremely high prevalence of cardiovascular disease seen in younger adults living with HIV.

Who leads patient care is often determined by which of the co-existing conditions is most complex to manage, thus defaulting to that specialist caregiver. The reality is that diverse expertise is required for effective management of multimorbid individuals. In low-income settings, the basic lack of healthcare workers poses the single largest barrier to effective healthcare implementation. In high-income settings, excellent vertically specialized healthcare systems for individual conditions usually exist with different providers for kidney disease, heart disease, diabetes, and mental health. Nevertheless, integrating care across many medical specialties and individual providers has proved challenging and evidence would suggest that healthcare systems are failing to deliver for these patients [5]. If success is to be achieved anywhere, of primary importance is a standardized approach to multimorbidity.

A plethora of differing approaches to measuring and calculating multimorbidity, based on the co-existence of several different conditions and combinations thereof, have been proposed by multimorbidity researchers over the years [6,7]. As a result of this heterogeneity, wide disparities exist in the reported prevalence of multimorbidity [8], as well as the cost implications, across the globe. Without a consistent approach to defining multimorbidity, determining those at highest risk and establishing true prevalence becomes nigh on impossible, the downstream effect of which is an inability for policy makers to direct adequate resources for either its prevention or effective management.

Approaching multimorbidity via individual conditions culminates in increased healthcare utilization and expenditure. Current data estimate that healthcare costs are significantly higher when treating multimorbid individuals and that, with certain combinations of co-existing conditions, costs may be higher still [9]. Excess expenditure has been attributed to a variety of factors including duplicated appointments and investigations across both primary and secondary care, and increased attendance at emergency departments [10], all without clear evidence of improvement to patients’ quality of life or physical health outcomes. Multimorbidity is a major driver of polypharmacy and the associated negative consequences for patients and healthcare systems including adverse drug events, hospital readmissions and even mortality [11]. Given the current global economic climate, maximizing the use of available resources should be a collective priority. A joined-up, multidisciplinary, structured approach to care for multimorbid patients is undoubtedly required and integration of care pathways which involve a diversity of specialist expertise are strongly supported in some circumstances [12]. Theoretically, primary care should serve to integrate diverse involvements by direct engagement with patients. However, considering the paradigm shift towards multimorbidity, arguments certainly exist in favor of ensuring that healthcare professionals obtain and nurture core generalist skills alongside specialist skills to facilitate complete and holistic caregiving in the face of multimorbidity [13].

The way clinical research is approached must also be carefully considered. Currently, evidence obtained through randomized controlled trials is considered poorly applicable to multimorbid patients because of strict inclusion and exclusion criteria applied to avoid confounding in these trials. In practice that means multimorbid patients receive treatments which have been shown to be very beneficial in isolated conditions but without an understanding of whether or how they may impact other conditions or treatments. Limited research has investigated the interplay between multimorbid diseases, and few trials have included multimorbid patients. Published articles are limited to those with cancer or focus on the mental health consequences of co-morbid illnesses such as depression and anxiety in cardiac disease [14]. Conversely, the very poor life expectancy of those with serious mental illness has been reported and investigated extensively, helpfully contributing to public health planning [15].

It is vital that researchers, clinicians, and policy makers re-think how research findings can be applied to real-world settings where multimorbidity exists in abundance. In LMICs, research should envision, design and test programs for multimorbid care led by health workers who are abundant, such as community health workers, peers, and volunteers. In HICs research should elucidate novel approaches to measuring multimorbidity and strengthening primary care of multiple chronic conditions, working towards truly human-centered care which integrates multiple treatment and care approaches. The UK NHS and other large healthcare systems should be well placed to characterize multimorbidity in HICs and provide platforms for research on interventions including drug sparing and care provision.

As is the case with most pandemics, the enormity of the problem is growing exponentially, and global action is required. The warning signs are there, and the trend will continue—the healthcare profession needs to be prepared and poised to act.

## Author Contributions

Wrote the first draft of the manuscript: PD. Contributed to the writing of the manuscript: TB CD PD. Agree with the manuscript’s results and conclusions: TB CD PD LGB. All authors have read, and confirm that they meet, ICMJE criteria for authorship.
